# Phototransduction and circadian entrainment are the key pathways in the signaling mechanism for the baculovirus induced tree-top disease in the lepidopteran larvae

**DOI:** 10.1038/s41598-018-35885-4

**Published:** 2018-12-03

**Authors:** Upendra Raj Bhattarai, Fengjiao Li, Mandira Katuwal Bhattarai, Abolfazl Masoudi, Dun Wang

**Affiliations:** State Key Laboratory of Crop Stress Biology for Arid Areas, Northwest A&F University, Yangling, Shaanxi 712100 P. R. China

## Abstract

The tree-top disease is an altered behavioral state, displayed by baculovirus-infected lepidopteran larvae, and characterized by climbing to an elevated position before death. The detailed molecular mechanism underlying this phenomenal behavior change has not been reported yet. Our study focused on the transcriptomic changes in the host larvae due to baculovirus infection from pre-symptomatic to tree-top disease stage. Enrichment map visualization of the gene sets grouped based on the functional annotation similarity revealed 34 enriched pathways in signaling mechanism cluster during LdMNPV induced tree-top disease in third instar *Lymantria dispar asiatica* larvae. Directed light bioassay demonstrated the positively phototactic larvae during tree-top disease and the gene expression analysis showed altered rhythmicity of the host’s core circadian genes (*per* and *tim*) during the course of infection emphasizing the role of Circadian entrainment and Phototransduction pathways in the process, which also showed maximum interactions (>50% shared genes with 24 and 23 pathways respectively) among other signaling pathways in the enrichment map. Our study provided valuable insights into different pathways and genes, their coordinated response and molecular regulation during baculovirus infection and also improved our understanding regarding signaling mechanisms in LdMNPV induced tree-top disease.

## Introduction

Parasite’s varied strategy of host behavior manipulation for their own utility maximization has been well documented in many species. These strategies utilize physiological, morphological or behavioral changes in the host through genetic, enzymatic, biochemical or immunological modification, which surface as a distinct aberrant phenotype^1,2^. Few noteworthy examples include amphipods manipulated by acanthocephalans or microsporidia^3–5^, hairworms infected by nematomorph^6–8^, ants infected by fungus^9^. Majority of these findings have highlighted one or more of many environmental clues like light, water, gravity as a major stimulus for the behavior alteration.

Behavior manipulation of lepidopteran larvae by baculoviruses had been in loop among researchers since 1891 when the first record of baculovirus epizootics in natural insect population was observed, though the causal agent of the disease was unknown then, the distinct altered behavior displayed by diseased larvae, migrating to and die at the top of the trees gave rise to the term tree-top disease (*Wipfelkrankheit*)^10^. This enhanced unidirectional locomotory activity before death induced by baculovirus in the infected larvae appears at the final stage of behavior alteration, whereas aberrant hyperactivity is apparent in the host insect at the initial stage^11–13^; and these two different altered behaviors are governed by different mechanisms in the baculovirus^14^. There have been extensive investigations for the triggering mechanisms in the virus for both of these behaviors. The viral gene protein tyrosine phosphatase (*ptp*) from *Bombyx mori* nucleopolyhedrovirus (BmNPV) and *Autographa californica* nucleopolyhedrovirus (AcMNPV) had been reported as the inducer of hyperactivity in their host larvae^11,15^, whereas *egt* gene from *Lymantria dispar* nucleopolyhedrovirus (LdMNPV) was found responsible for tree-top disease in *L*. *dispar* larvae^16^ but not in other lepidopteran-baculovirus interactions^17^. Amid in-depth molecular and genetic research and understanding of baculovirus for induction of altered behaviors; studies for the investigation of host’s response and coordinated mechanism of genetic and molecular interactions within the host larvae producing or reacting to the impetus for either kind of changed behavior is very limited. Transcriptomic study of BmNPV infected *B*. *mori* larval brain and LdMNPV infected *L*. *dispar* larvae during hyperactivity provided useful insights on the transcriptomic changes in the host larvae^12,13^, but there are no any such studies focused on baculovirus induced tree-top disease. van Houte and colleagues^18^ reported positive phototactic response of *Spodoptera exigua* larvae infected with SeMNPV during tree-top disease. They further investigated the temporal requirements for light and found that light in particular time-frame during infection is required to induce tree-top disease^19^. But no underlying molecular mechanism has been studied yet.

In this study, the third instar Asian gypsy moth larvae (*L*. *dispar asiatica*) and its pathogenic baculovirus LdMNPV were used as a model system to study the transcriptomic changes in the host larvae during tree-top disease. Initially, the timing of tree-top disease was determined with no molting from infection to death to remove the molting dependent gene expression biases of larvae for subsequent analysis. Then a genome-wide high-throughput transcriptome sequencing was carried out from the samples at specified time points. Transcriptomic analysis was focused on deciphering the coordinated mechanisms of the differentially expressed host’s genes (DEGs) through enrichment map visualization of enriched gene sets, followed by different gene expression studies and bioassays. This study provides the foundation for a better understanding of the molecular mechanisms within the host larvae from infection period to the tree-top disease stage and also signifies the importance of environmental clues as host impetus in the process.

## Results and Discussions

### Locomotion assay

**Figure 1:**
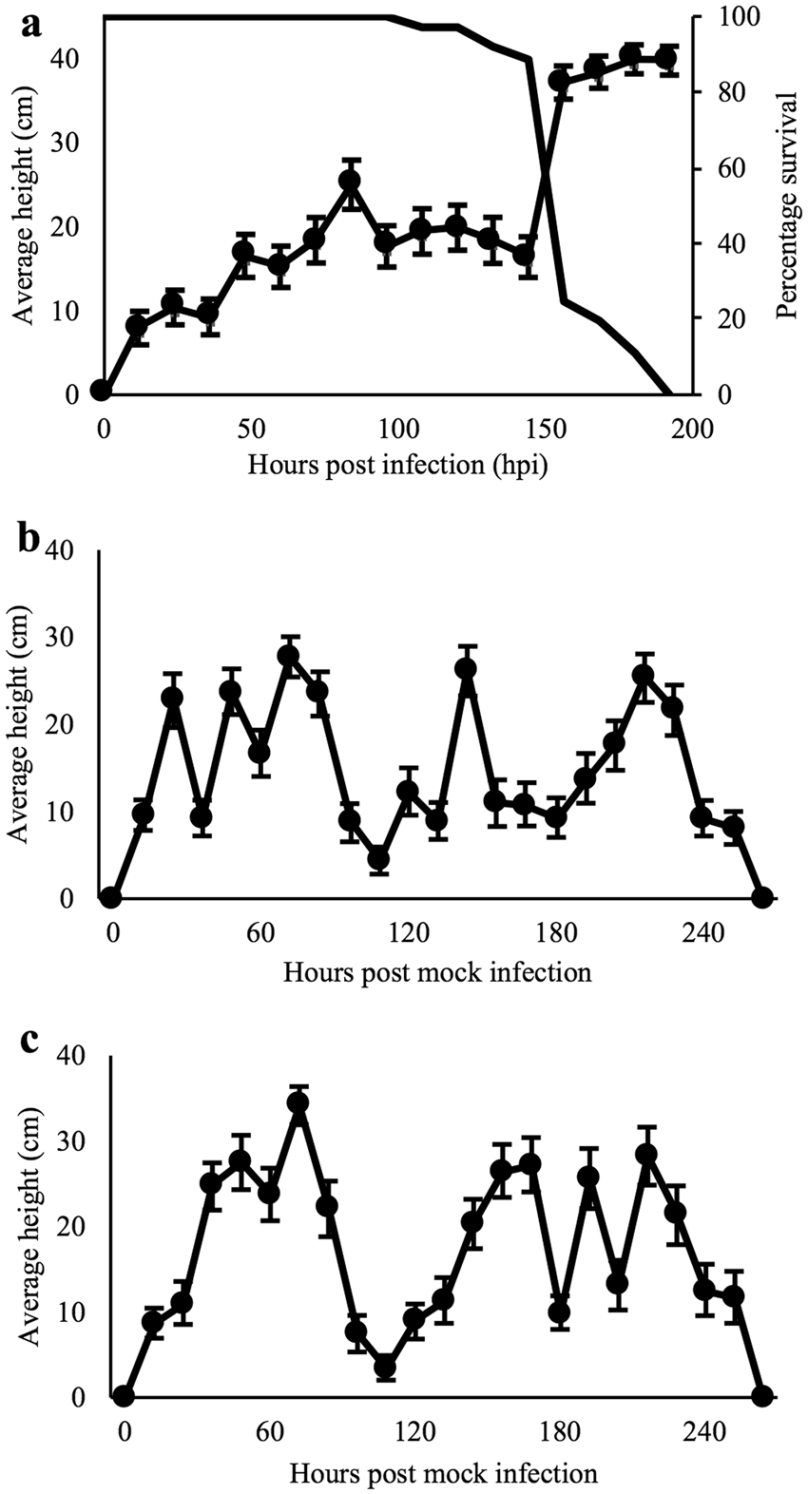
Larval climbing behavior during the course of time, (**a**) relationship between vertical position of the *L*. *dispar* third instar larvae and their mortality exhibiting tree-top disease induced by LdMNPV, 14: 10 (L: D hours) (n = 36), line with closed circles represents average height of larvae in cm (left Y-axis), the other black line represents percentage survival of the larvae (right Y-axis); (**b**) climbing behavior of mock infected larvae at photoperiod of 14: 10 (L: D hours) (n = 39); (**c**) climbing behavior of mock infected larvae at photoperiod of 0: 24 (L: D hours) (n = 31). The x-axis represents hour post infection and right Y-axis represents the average vertical position of the larvae in cm and error bars represents standard error mean (SEM).

We conducted a series of behavioral assays to determine the time point for the exhibition of tree-top disease by *L*. *dispar* third instar larvae upon LdMNPV infection. Virus-infected larvae showed a clear climbing response before death. They were found ascending rapidly in the column after 144-hour post infection (hpi) (mean height: 17.53 ± 3.34 cm at 144-hpi, 34.59 ± 3.66 cm at 156 hpi; Fig. 1a) (Wilcoxon signed-rank test, Z = −4.703, *P* < 0.0001). Survival was 90% at 144-hpi, which decreased to 35% at 156-hpi (Fig. 1a). There was a significant negative correlation between height and the survival percentage of the larvae (Kendall’s tau coefficient = −0.612, *P* = 0.001). Uninfected larvae showed no difference for climbing behavior between light/dark (14 L: 10 D) or complete dark (0 L: 24 D) conditions. These larvae showed several climbing peaks irrespective of light conditions and descended to the bottom before pupating (Fig. 1b,c). A similar observation had been reported for the induction of tree-top disease in *Spodoptera exigua* third instar larvae infected by SeMNPV^18,20^.

This experiment identified 144-hpi as a time point for the induction of tree-top disease, where we have baculovirus infected sample (NPV_6d). In addition, we obtained baculovirus treated samples (NPV_3d) as a pre-symptomatic stage and mock treated sample (CK_3d) as a control at 48-hpi for RNA-Seq study. All the samples were from the third instar of the larval developmental stage.

### Sequencing and *de novo* assembly of the *L*. *dispar* transcriptome

A comprehensive larval reference transcriptome of *L*. *dispar* was generated with nine Illumina RNA-Seq libraries with an average insert size of 175 bp. Illumina deep sequencing of the libraries yielded 443,082,526 raw paired-end reads, which were quality filtered including the removal of viral transcripts. No significant number of viral transcripts were observed at NPV_3d samples but the removed viral reads at NPV_6d samples accounted for a total of 5,730,667 reads (3.95%) producing 427,770,700 total clean reads (Table 1). *De novo* assembled reference transcriptome resulted in 271,496 assembled transcripts with a minimum length of 201 bp and a N50 value of 1,394 bp and 169,188 unigenes with the minimum length of 201 bp and N50 value of 1,797 bp.

**Table 1:** Statistical summary of Illumina sequencing data, quality filter and mapping to *de novo* assembly.

Attributes	CK_3d1	CK_3d2	CK_3d3	NPV_3d1	NPV_3d2	NPV_3d3	NPV_6d1	NPV_6d2	NPV_6d3
Raw reads	45817380	52466998	53506222	46546858	56356502	43202066	42139796	48833836	54212868
Clean reads	44151034	50633296	51590402	44902496	54380374	42042592	40678616	47104364	52287526
Clean bases	6.62 G	7.59 G	7.74 G	6.74 G	8.16 G	6.31 G	6.1 G	7.07 G	7.84 G
Mapped reads	32346074 (73.26%)	42938242 (84.80%)	40274286 (78.07%)	36108506 (80.42%)	44092228 (81.08%)	33790780 (80.37%)	31696132 (80.96%)	39121438 (83.05%)	30358798 (81.04%)

### Functional annotation and differential gene expression analysis

The biological meaning of the expression data was derived from their functional annotation to seven different databases. The annotation resulted in a total of 61,053 (36.08%) unigenes annotated in at least one database and 3.19% of unigenes annotated in all seven databases, including 22.79% in Nr, 21.31% in SwissProt, 24.66% in Pfam, and 10.42% in KO database (Table S1). Furthermore, the distribution and frequency of homology search resulted in the highest match (37.7%) with sequences of silkworm (*Bombyx mori*) (Fig. S1).

**Figure 2:**
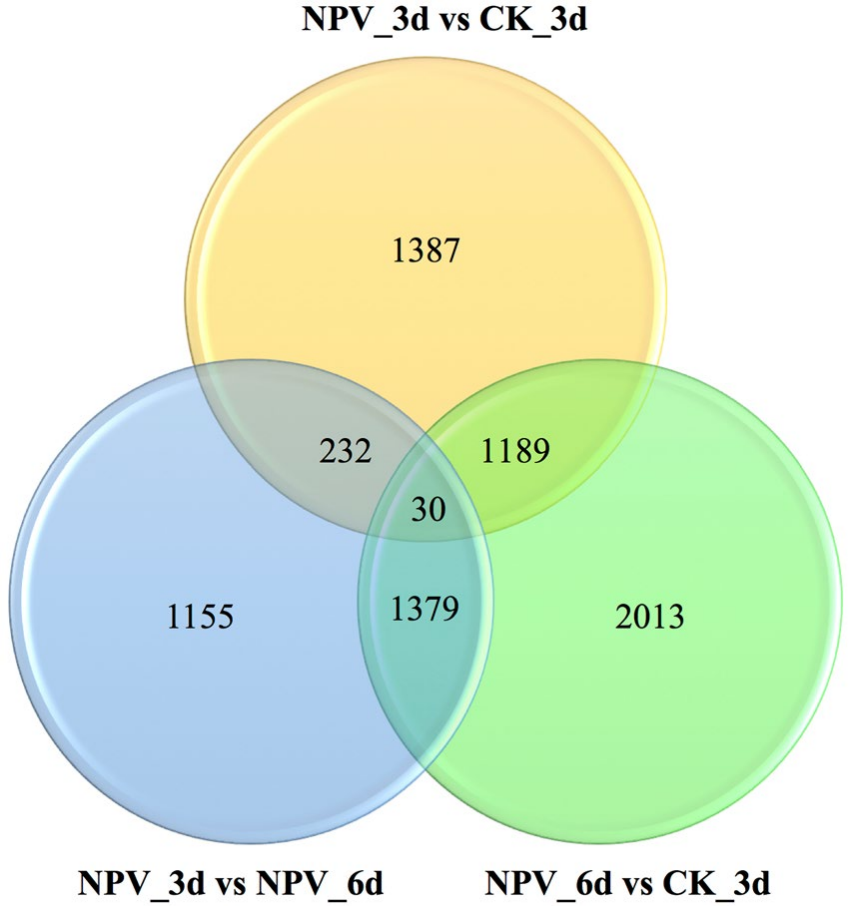
Venn diagrammatic representation of differential gene expression analysis in pairs showing the number of significantly (*P* ≤ 0.05) differentially expressed genes between NPV_3d Vs CK_3d, NPV_6d Vs CK_3d, NPV_6d Vs NPV_3d.

Mapping of RNA-Seq reads from each library to *de novo* assembled reference transcriptome revealed the highest match of 84.80% from the CK_3d2 sample, whereas lowest mapped reads were from CK_3d1 sample with 73.26% (Table 1). Differential expression analysis resulted in 30 commonly differentially expressed unigenes between pairwise comparison (Fig. 2). Similarly, 1,160 unigenes were up-regulated and 1,678 unigenes were down-regulated in NPV_3d vs CK_3d (Fig. S2a); 891 unigenes were up-regulated and 1,905 unigenes were down-regulated between NPV_3d and NPV_6d (Fig. S2b) and 2,924 unigenes were up-regulated and 1,687 were down-regulated between NPV_6d and CK_3d (Fig. S2c).

### GO, KEGG, and KOG enrichment analysis

The functional categorization of the differentially expressed unigenes with gene ontology (GO), Kyoto Encyclopedia of Genes and Genomes (KEGG) and euKaryotic Orthologous Groups of proteins (KOG) allowed for the unified biological explanation of genes and gene products attributes across all species. Annotation in the GO database resulted in the total of 112,176 unigenes in biological process category; 48,970 unigenes in molecular function category; and 68,844 unigenes in the cellular component category (Fig. S3a). The GO enrichment analysis of unigenes for NPV_3d vs CK_3d showed extracellular region as the most enriched GO term with 121 unigenes among significantly (corrected *P* < 0.05) enriched GO terms (Fig. S4a). Similarly, for NPV_3d vs NPV_6d, metabolic process in the biological process category was the most enriched with 1,082 unigenes, and cell and cell part in the cellular component category with 610 unigenes. Similarly, catalytic activity in molecular function category with 844 unigenes was the most enriched among significant GO terms (Fig. S4b). Furthermore, between NPV_6d and CK_3d, metabolic process and organic substance metabolic process in biological process category with 1,760 and 1,443 unigenes respectively; macromolecular complex in cellular component and catalytic activity in molecular function category with 642 and 1,393 unigenes respectively were the most enriched GO terms (Fig. S4c).

KEGG is a collection of manually curated databases dealing with genomes, biological pathways, diseases, drugs and chemical substances. KEGG enrichment analysis can identify significantly enriched metabolic pathways or signal transduction pathways associated with differentially expressed genes compared with the whole genome background. A total of 6,585 unigenes were annotated in metabolism category; 5,379 in organismal system category; 2,911 in genetic information processing category; 2,772 in environmental information processing category and 2,767 in cellular processes category. Among subcategories, the signal transduction was the most enriched with 2,317 unigenes followed by the endocrine system (1,390 unigenes) (Fig. S3b). From the pairwise comparison between NPV_3d vs CK_3d: ECM-receptor interaction, protein digestion, and absorption were significantly down-regulated, whereas several metabolism pathways including longevity regulating pathway-multiple species were significantly up-regulated (Fig. S5a,b). Similarly, ribosome, parkinson’s disease, huntington’s disease pathways showed significant down-regulation in NPV_3d vs NPV_6d but viral myocarditis was significantly up-regulated (Fig. S5c,d). Furthermore, the comparison between NPV_6d vs CK_3d revealed down-regulation of pathways like lysosome, focal adhesion, protein digestion and absorption, amino sugar and nucleotide sugar metabolism but upregulation of ribosome, parkinson’s disease, oxidative phosphorylation, huntington’s disease, and citrate cycle pathways (Fig. S5e,f).

Unigenes were also defined for their orthologous functions using KOG database. The general function prediction only group possessed the highest proportion (5,944 unigenes, 22.54%) followed by signal transduction mechanisms with 3,620 unigenes (13.73%) and posttranslational modification, protein turnover chaperons with 2,448 unigenes (9.28%) (Fig. S6).

### Gene set enrichment analysis (GSEA) and Enrichment map visualization

**Figure 3:**
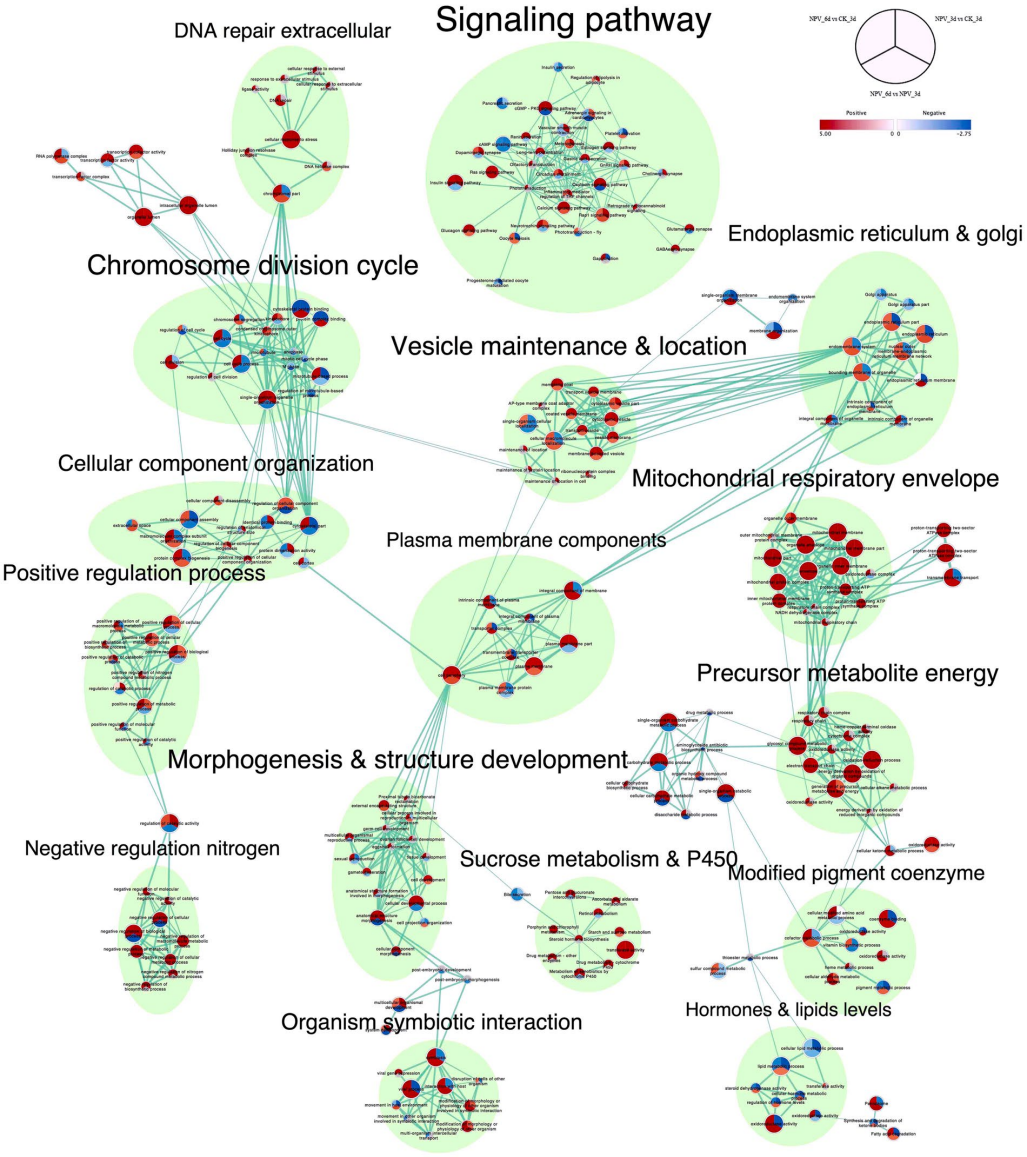
Enrichment Map visualization of the ranked GSEA^62^ results obtained for comparative analysis between each permutation of CK_3d, NPV_3d, and NPV_6d. Each node represents enriched GO/KEGG term, node size is proportional to the number of genes in each node and is divided into three parts representing different pairwise comparisons as illustrated in figure legend; node color gradient represents the normalized enrichment scores (NES). The thickness of edges represents the richness of shared genes between connected nodes. Different clusters represent the biological meaning of involved pathways as depicted by AutoAnnotate plugins of Cytoscape, font size of the cluster label is proportional to the cluster size. Complete map including the nodes which were not part of any clusters is presented in Supplementary Fig. S9.

**Figure 4:**
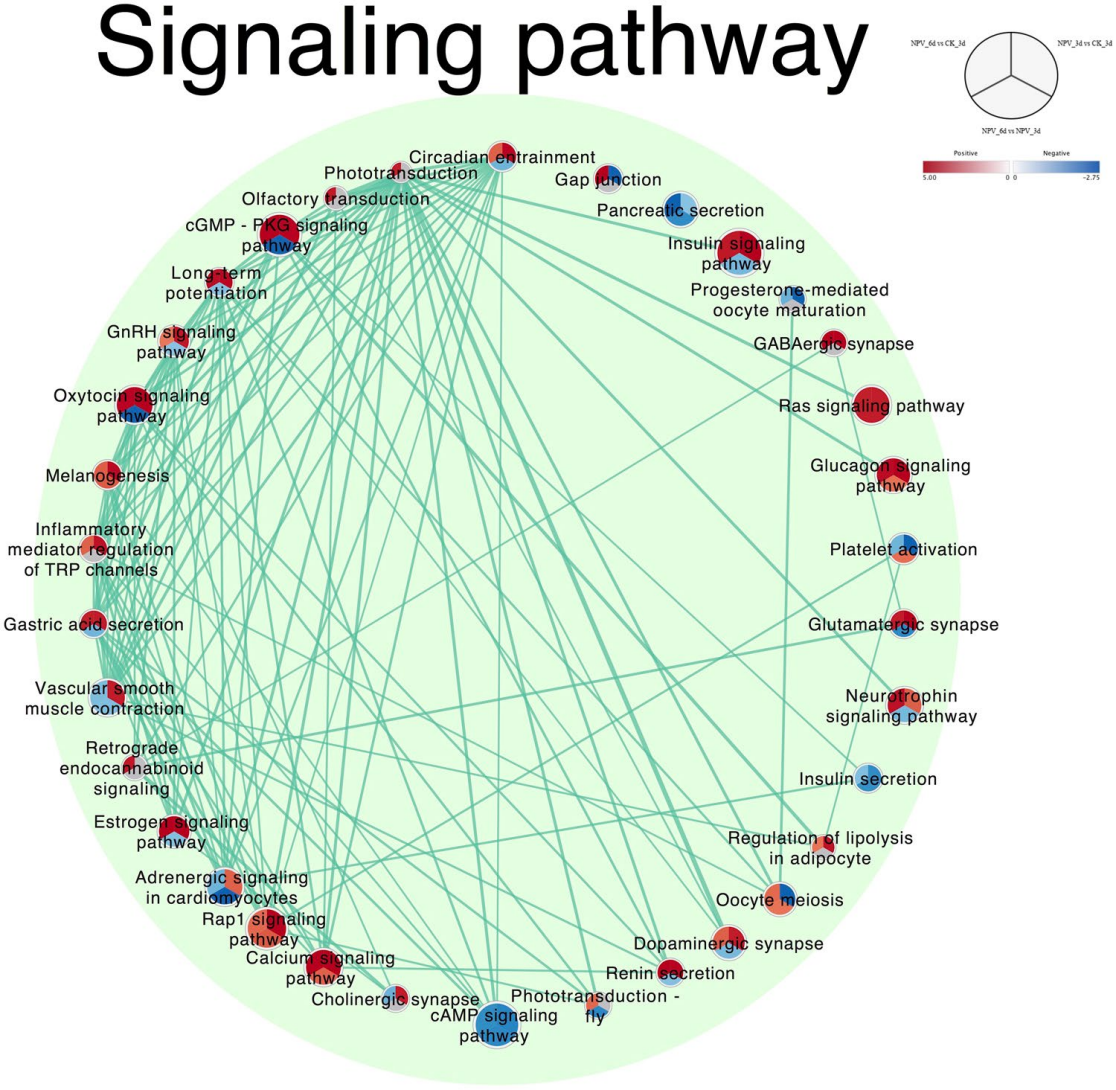
Signaling pathway cluster of enrichment map extracted from Fig. 3.

Gene set enrichment analysis has been proposed and successfully used as a means to identify meaningful biological gene sets with impact on the risk of diseases and various phenotypes^21^.Gene sets here refers to the group of genes sharing common functions or operating in the same pathways. We tested for the functional enrichment of gene sets among differentially expressed genes in our dataset through GSEA and visualized their interactions and differential representations through enrichment map to identify the biological processes involved in the tree-top disease. Such an enrichment analysis can combine single-gene effects to a meaningful biological pathways^22^. Normalized enrichment scores were used to graphically visualize the functional enrichment map (Fig. 3, 4 and S7). 16 different clusters of gene sets were produced through auto-annotation and clustering, each of them representing their own broad functional activities (Fig. 3). Among all the clusters signaling pathway cluster was the biggest with 34 gene sets followed by chromosome division cycle cluster with 17 gene sets.

The signaling mechanism holds a significant hand for the behavioral decisions and different genes and proteins in different signaling cascades are organized into complexes, accounting for the efficacy and specificity of the pathways involved in response to particular extracellular stimuli^23,24^. So, we focused our further analysis on the prominent pathways and their interactions with one another within signaling pathway cluster. The signaling pathway cluster consisted of 34 enriched pathways and was re-laid out in degree sorted circle layout at >0.6 overlap coefficient to make it easy to visualize the involvements and interactions of each node within the cluster (Fig. 4). Circadian entrainment, Phototransduction and, Olfactory transduction pathways were with the highest interactions (24, 23 and, 22 connected pathways respectively) at default overlap coefficient of >0.5.

Circadian gating of sensory information seems to be a widespread phenomenon^25,26^. It has been found to be a key modulator in virulence of pathogens^27^, immune systems^28,29^ as well as in the host-parasite interactions^30^ but no study has thus far looked at how their expression changes over the course of baculovirus infection leading to tree-top disease. Upregulation of circadian entrainment pathway from CK_3d to NPV_3d and downregulation from NPV_3d to NPV_6d but still maintaining upregulation at NPV_6d compared to CK_3d showed its clear involvement from pre-symptomatic to tree-top disease induction stage. The maximum cross talks of circadian entrainment pathway having more than 50% overlapping genes with other 24 pathways in the cluster hinted the need for further analysis of the involved genes in this pathway.

Similarly, the phototransduction mechanism absorbs photons and convert them to electrical signals triggering various functional systems in the organism^31^. The phototransduction and olfactory-transduction showed the highest upregulation at NPV_6d compared to CK_3d but no changes at NPV_3d stage signified their involvement only at tree-top disease induction stage. Thylakoid part, membrane, and photosystem gene sets showed downregulation from CK_3d to NPV_3d and steeper downfall to NPV_6d supporting the active involvement of phototransduction system during the altered behavioral response. Phototaxis had previously been recognized to play an important role to cause tree-top disease in baculovirus infected lepidopteran larvae^18,19^ but its mechanistic involvement in genetic level was unanswered. Our analysis revealed that phototransduction mechanism in the host insect plays an active role in the signaling of before death climbing behavior of the LdMNPV infected *L*. *dispar* third instar larvae. Furthermore, gene sets in DNA repair extracellular cluster showed upregulated response of larvae to external and extracellular stimulus during tree-top disease stage (NPV_6d) indicating the involvement of external stimulus.

Similarly, majority of the gene sets involved in precursor metabolites energy cluster like oxidation-reduction process, generation of precursor metabolites and energy, glycosyl compound metabolic process energy derivation by oxidation of organic compounds, electron transport chain were upregulated continuously from CK_3d to NPV_3d and to NPV_6d, whereas respiratory chain complex, oxidoreductase activity, cellular alkane metabolic process, energy derivation by oxidation of reduced inorganic compounds, oxidoreductase activity, heme-copper terminal oxidase activity and cytochrome complex were only upregulated in NPV_6d compared to NPV_3d and CK_3d. All the gene sets in mitochondrial respiratory envelope cluster showed upregulation from CK_3d to NPV_3d and continue to NPV_6d except respiratory, oxidoreductase and NADH dehydrogenase complex, which were upregulated only in NPV_6d compared to CK_3d signifying the increase in energy demands by the larvae during tree-top disease stage.

### Validation by quantitative RT-qPCR

Expression of nine randomly selected DEGs: *jun*, *ddc*, *gabarap*, *glul*, *myh6*, *actb-g1*, *hspa-1*, *pepck*, *ant* was again tested using real-time PCR to check the validity of the RNA-Seq data. The expression profiles showed consistency between two methods and had a positive linear correlation (Pearson correlation: NPV_3d vs CK_3d, R^2^ = 0.8983, *P* = 0.0001; NPV_6d vs NPV_3d, R^2^ = 0.8654, *P* = 0.0003; NPV_6d vs CK_3d, R^2^ = 0.9192, *P* < 0.0001) (Fig. S8a–c).

### Expression analysis of circadian genes

**Figure 5:**
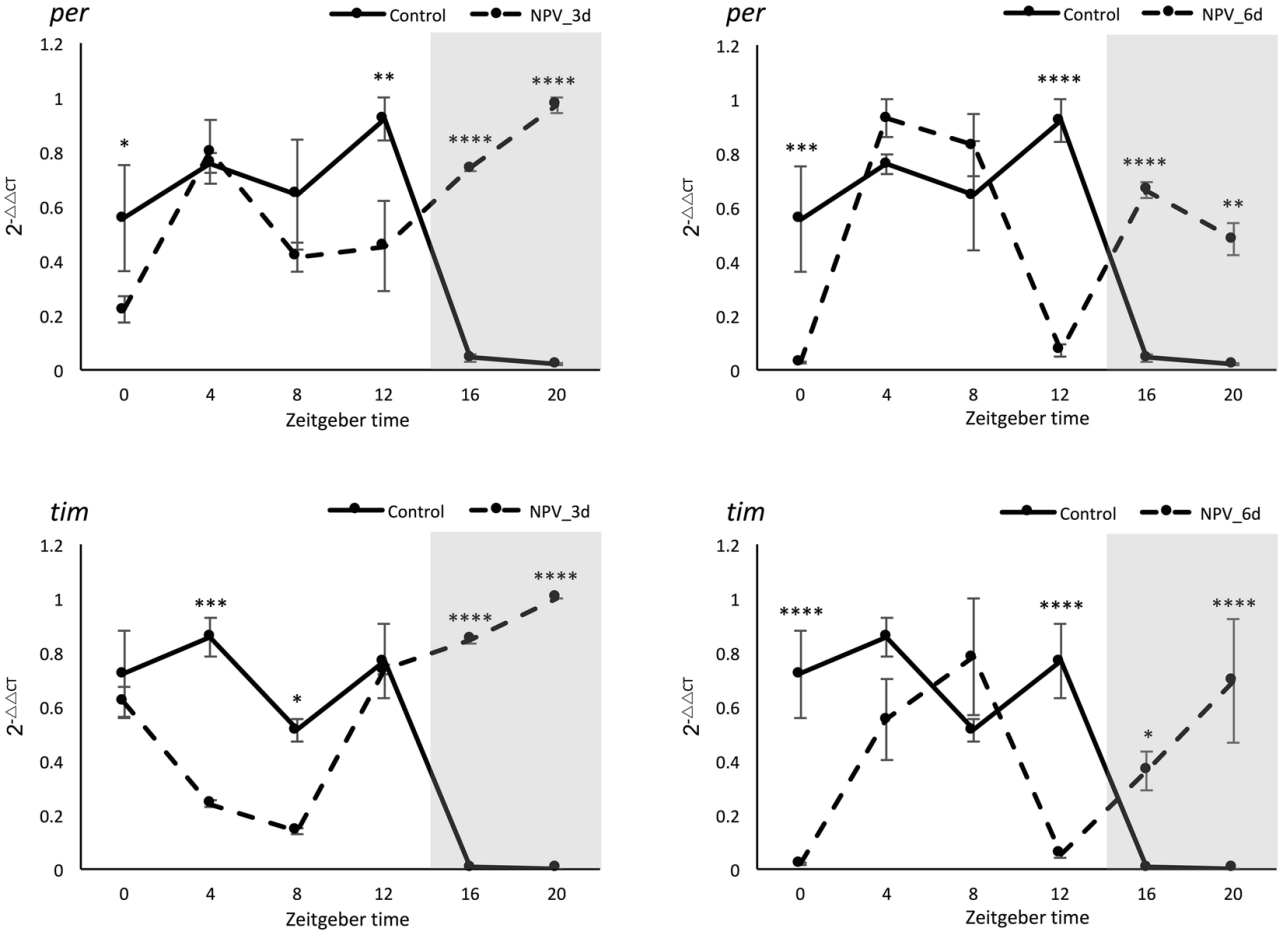
Rhythmic expression of *per* and *tim* genes during 24 hours in *L*. *dispar* third instar larvae. Gene expression data regards qPCR analysis of whole insect body in three experimental groups (Control, 3-dpi, and 6-dpi of baculovirus infection) sampled at each 4-hour represented by *Zeitgeber* time (0 to 20), starting from the time lights came on (ZT0) (represented by X-axis); the shaded portion in the graph represents the dark hours (ZT14 – ZT24). Y-axis represents the expression values in 2^-ΔΔCT^, normalized with the expression of the housekeeping gene and calibrated with the lowest normalized CT value among samples from the same treatment and day. Line graphs represent the mean expression values with error bars for standard error mean, the experiment was conducted with three independent biological replicates and three technical replicates for each sample. Expression data were compared with the control group and *, **, ***, ****, denotes significance at 5, 1, 0.1 and 0.01%, respectively (Dunnett’s multiple comparisons test).

The expression profiles of core circadian genes (homologs for *per* and *tim*) during a complete circadian cycle (every 4 hours) at 3 and 6-dpi of virus infection was evaluated against the control group in 14 L: 10 D (light: dark) photoperiodic setting. Two-way ANOVA analysis revealed no significant effect of the experimental groups on *per* gene expression. However, highly significant (*P* < 0.0001) effect of both zeitgeber time and zeitgeber time × experimental groups interaction was observed on *per* gene expression. In the meantime, significant (*P* = 0.0259) effect of experimental groups and highly significant (*P* < 0.0001) effect of zeitgeber time × experimental groups interaction were observed on *tim* gene expression but the effect of zeitgeber time was non-significant. Dunnett’s multiple comparison tests conducted for each time points on each experimental group against control are presented in Fig. 5. Expression pattern of both the genes suggests that their expressions were high during light hours and diminished during dark hours in control samples, which is a regular rhythm of the genes in the negative loop of circadian cycle^32^. But their expression changed drastically with continuous expression throughout 24 hours irrespective of light/dark cycle in NPV_3d samples. This further changed in NPV_6d samples exhibiting two peaks and two low phases within the 24-hour period (Fig. 5). Circadian entrainment pathway from KEGG database leading to phase advance in circadian rhythm is shown in the Fig. S9 with expression profiles of different annotated genes from our RNA-Seq data. where most of G-proteins showed upregulation at NPV_6d compared to NPV_3d and CK_3d.

The behavioral light response under circadian regulation in larval phototaxis had been reported in *Drosophila* larvae through clock genes mutant study; larvae with *clk* and *cyc* mutation showed constant low-level expression of *per*, *tim* and other similarly regulated genes exhibiting high photophobic response. Whereas *per* and *tim* null mutants had depressed *clk* and *cyc* activity leading to constitutively high levels of *per* and *tim* transcription and were less responsive to light compared to photophobic wild-type larvae^33^. While our findings do concur with these findings as the overexpression of *per* and *tim* mRNA during tree-top disease could have affected the phototactic response of third instar gypsy moth larva.

Furthermore, involvement of Pigment-dispersing factors (PDFs) widely distributed and coexisted with *per* in insect optic lobes and brain are found to be actively involved in regulating the locomotor activity in drosophila and cricket, *Teleogryllus commodus*^34,35^ as well as in various complex behavioral and physiological traits in insect including geotaxis^36^, circadian phase shifting^37,38^ and in regulation of ecdysone biosynthesis via G-protein couple receptors and also through extracellular Ca^2+^ mediated pathways^39,40^. In this context, significant differential regulation of *per* gene, alteration of circadian rhythm, significant upregulation of extracellular Ca^2+^ and guanine nucleotide-binding proteins and positive phototactic response of larvae during tree-top disease may have indicated the possible involvement of PDFs thereby regulating 20-hydroxyecdysone (20E), which was found to be a major inducing factor of tree top disease^16,41^.

### Candidate DEGs involved in sensory perception

With the progression of the infection, genes related to sensory perception involved in phototransduction and olfactory transduction pathways showed remarkable differential expression. In our study, 54 unigenes were annotated in phototransduction pathway, 117 in the phototransduction-fly pathway and 87 in olfactory transduction pathway, among which 14 genes in phototransduction and 10 in olfactory transduction pathways were differentially expressed between at least two samples in pairwise comparisons (Table 2). DEGs in the phototransduction pathway involved ACTB_G1 (5 unigenes within), two of the unigenes showed continuous upregulation (Log2FC > 7) at NPV_6d compared to CK_3d. While the other 3 were downregulated by (Log2FC < −1) at NPV_6d vs CK_3d. These actin-binding proteins are required not only to maintain cell-shape and changes but also in motility, different taxis, intracellular transport processes and signal transduction^42,43^. They are important structural and functional part of arthropod phototransduction mechanisms^44^. Different regulatory proteins from the actin cytoskeleton are involved in the various processes including phototaxis, where gelatin factor (GRP125) was found to alter phototactic behavior^42,45^.

**Table 2:** Expression analysis results in log2fc for all the differentially expressed genes in pairs involved in phototransduction and olfactory transduction pathways.

Pathways		Gene ID	Name	Log2FC*		
				NPV_3d vs CK_3d	NPV_6d vs NPV_3d	NPV_6d vs CK_3d
	Phototransduction Pathway	Cluster-4931.0	ACTB_G1	2.7036	4.2239	7.3509
		Cluster-5147.0	ACTB_G1	3.5951	2.6654	7.1635
		Cluster-48789.68851	ACTB_G1	−1.8186	1.2975	−0.56918
		Cluster-48789.66190	ACTB_G1	0.66381	−1.6536	−1.0009
		Cluster-48789.68978	ACTB_G1	0.76394	−1.7604	−1.0069
		Cluster-48789.84659	ITPR1	−5.3821	6.8055	1.3776
		Cluster-48789.101075	PLCB	2.08	−1.1866	0.85293
		Cluster-48789.40932	PPEF, PPP7C	−2.4871	0.34958	−2.1482
		Cluster-48789.40934	PPEF, PPP7C	0.76058	−6.1387	−6.3776
Olfactory transduction pathway		Cluster-48789.103227	CALM	3.7725	−0.60401	3.1591
		Cluster-864.0	CALM	—	6.1581	6.9904
		Cluster-3416.0	CALM	3.2773	5.1328	9.0282
		Cluster-48789.66595	CALM	2.9969	−0.57756	2.3885
		Cluster-3970.0	GNB1	2.5994	5.121	7.6645
		Cluster-48789.23761	ARRB	1.4536	1.1933	2.6346
		Cluster-40264.0	PKA	1.06	3.5444	4.621
		Cluster-48789.89941	PRKG1	−1.0939	−5.0692	−7.1632
		Cluster-48789.62079	PRKG1	−7.9331	5.1179	−2.9736
		Cluster-50495.0	SLC24A4, NCKX4	−1.2354	−2.3361	−3.5807

Note: The overlapped rows between pathways represents differentially expressed genes involved in both pathways.

*Negative sign in the log2fc value indicates downregulated and vice versa.

Four DEGs were annotated to calmodulin and showed continuous upregulation up to Log2FC > 9 with the infection progression. Similar response of calmodulin genes in other organisms like *Physarum polycephalum*, *Dictyostelium* and *Chlamydomonas reinhardtii* where calcium fluxes were involved in sensory transduction of phototactic behavior have been reported^46–49^. Other DEGs in the phototransduction pathway included guanine nucleotide-binding protein, which was significantly upregulated in NPV_6d vs CK_3d (Log2FC = 7.66). These are heterotrimeric signaling G-proteins for G-protein-coupled receptors (GPCRs), which mediate responses to the wide range of extracellular stimuli. Their upregulation implies higher light sensation^50,51^. In some organisms like *Dictyostelium* where an extensive study on phototaxis, chemotaxis has been carried out suggest that G-proteins are actively involved in phototaxis movement and their inhibition will impair phototaxis effect^52^.

The serine/threonine-protein phosphatases with EF-hands were downregulated with the disease progression. In *Drosophila*, these novel phosphatases are encoded by retinal degeneration C (*rdgC*) gene in the visual systems and are required to prevent light-induced retinal degeneration. Loss of *rdgC* function causes a severe defect in the Ca^2+^ dependent termination of the light response^53,54^, again these receptor phosphatases are involved in the regulation of G protein-coupled signaling cascades through calmodulin-dependent protein phosphatases^55–57^. These shreds of evidence signify the role of GPCRs cascade operating through calcium flux regulation as a major regulator for positively phototactic larvae before death. All the calmodulin and guanine nucleotide-binding protein unigenes in the phototransduction pathway were shared with olfactory transduction pathway (overlapped rows for two pathways in Table 2).

Similarly, beta-arrestin and protein kinase A in olfactory transduction pathway showed continuous upregulation from CK_3d to NPV_3d and to NPV_6d whereas, cGMP-dependent protein kinase 1 and solute carrier family 24 showed downregulation with the progression of the infection.

### Directed light bioassay

**Figure 6:**
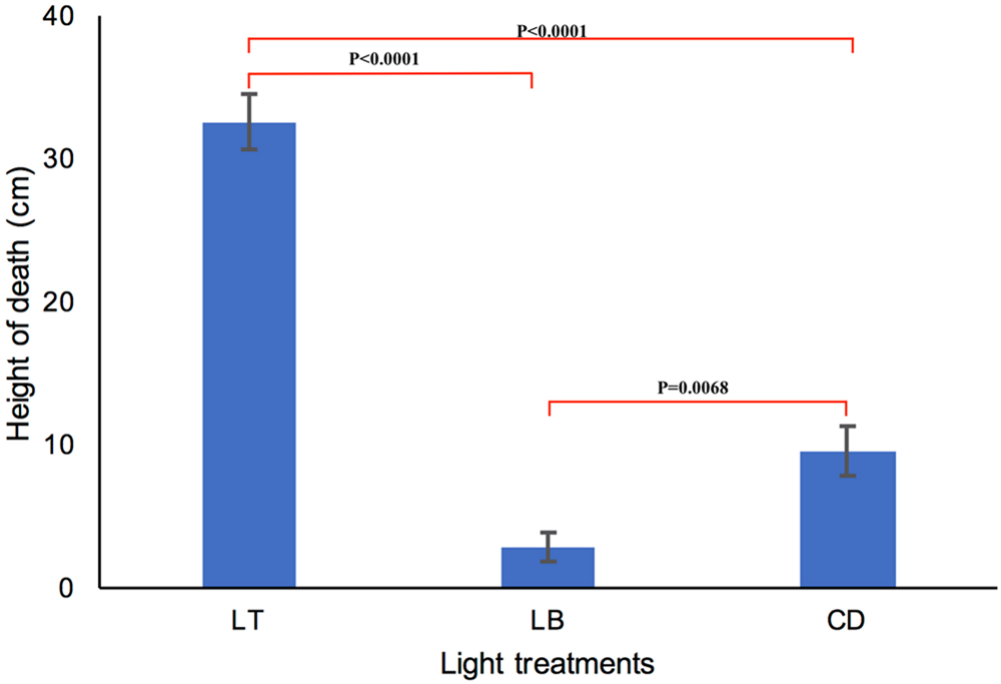
Positive phototactic response during tree-top disease induced by LdMNPV in third instar *L*. *dispar* larvae. Y-axis represents the average height of death in cm. X-axis represents different light treatments, Light from Top (LT) (Rep 1 and 2, n = 31; Rep 3 n = 32), Light from Bottom (LB) (Rep 1, n = 28; Rep 2 and 3, n = 36;), Complete dark (CD) (Rep 1, n = 29; Rep 2, n = 33; Rep 3, n = 32). *P*-values show the significance level in pairwise comparisons (Dunn’s multiple comparison tests). Error bars represent SEM.

The directed light bioassay revealed a strong positive phototactic response of the *L*. *dispar* third instar larvae during LdMNPV induced tree-top disease. Larvae subjected to light from the top died at significantly higher position (31.4 ± 1.6 cm) compared to the larvae exposed to light from the bottom (2.30 ± 2.04) (*P* < 0.0001, Dunn’s multiple comparison test) and to the larvae kept in complete dark (9.43 ± 0.03) (*P < *0.0001, Dunn’s multiple comparison test). Furthermore, the larvae exposed to light from the bottom died at the significantly lower position compared to those kept in the complete dark (*P* = 0.0068, Dunn’s multiple comparison tests) (Kruskal Wallis test across three groups K = 93.132; df = 2, P < 0.0001) (Fig. 6). This result is in accordance to the findings from van Houte, *et al*.^18^, where similar phototactic response during tree-top disease stage in *S*. *exigua* third instar larvae infected with SeMNPV was observed. This finding further strengthens the importance of the phototransduction pathway in tree-top disease as suggested by our GSEA analysis and states that LdMNPV infected *L*. *dispar* third instar larvae are positively phototactic during tree-top disease phase.

## Conclusion

In summary, GSEA of differentially expressed genes in diverse gene sets and functional annotations with an enrichment map visualization from *de novo* transcriptome analysis generated a thorough, yet concise picture of the underlying functional host’s mechanism through the progression of infection starting from pre-symptomatic to tree-top disease stage. The result provided broad insights on molecular regulation, pathways and their interactions involved in baculovirus infection process leading to tree-top disease and suggested a probable role of circadian entrainment, and phototransduction mechanism of the infected host larvae in the process. Gene expression analysis of core circadian genes in the negative loop and directed light bioassays further emphasized their role as key pathways in tree-top disease induction in *L*. *dispar* larvae.

## Materials and Methods

### Insects and virus

Eggs of *L*. *dispar asiatica* were obtained from Chinese Academy of Forestry, Beijing, China. Eggs were hatched and larvae were reared on the artificial diet under following conditions: temperature 26 ± 1 °C, relative humidity 60 ± 5% and a photoperiod of 14 L: 10 D (Light hours: Dark hours). Wild-type *L*. *dispar* nucleopolyhedrovirus (LdMNPV) obtained from the same source were reproduced in 4^th^ instar *L*. *dispar* larvae and stored at 4 °C until further use.

### Locomotion assay

Newly molted third instar larvae were starved for 24 hours and fed with 2 × 10^10^ OBs/Larva of LdMNPV using modified droplet feeding method^13^. Briefly, the prepared virus stock was mixed with equal volume of 10% sucrose solution with green colored food dye (Shaanxi TOP Pharm Chemical Co., Ltd, China) and 2 µl of inoculants prepared were placed at the bottom of the 1.5 µl Eppendorf centrifuge tubes. Each larva was guided inside those centrifuge tubes placed upside down. By 5 minutes, most larvae finished drinking the provided virus suspension by climbing up in the tube. Then each of them was transferred individually in a mesh wire column of 6 cm diameter and 45 cm height, with wire mesh lid on the top and food placed at the bottom (approx. 3 cm^3^). Photoperiod and environmental conditions were provided as they were reared upon. 15 W led lights at a distance of 30 cm from the column lid were used for luminescence during day condition. The vertical position of the larvae on the column was measured twice a day after infection until they were dead or pupated. Death from baculovirus infection was confirmed by screening for liquefaction of the larvae after death and death counts were recorded every day and those did not die of the virus were excluded from the analysis. A similar experiment was also carried out with mock-infected larvae.

### Sample preparation for RNA sequencing

Approximately 50 newly molted third instar larvae were starved for 24 hours and virus suspension (2 × 10^10^ OBs/Larva) was fed thereafter by droplet method as above. Each larva was then transferred to the 24 well plastic insect rearing box (12×6×2.5 cm^3^) in an individual well, supplemented with artificial diet (1 cm^3^). For control samples, the same volume of 10% sucrose solution with food dye was provided the same way. Whole insect samples at 3 and 6-dpi for baculovirus treated (NPV_3d and NPV_6d respectively) and at 3-dpi for mock-infected (CK_3d) were snap frozen in liquid nitrogen and then stored at −80 °C until further analysis. Samples were taken with three independent biological replicates at each time points and sequenced individually.

### RNA isolation cDNA library construction and Illumina sequencing

Total RNA was extracted from different samples using TRIzol reagent (Invitrogen) following the manufacturer’s protocol. The quality and quantity were assessed using 1% agarose gels, NanoPhotometer® spectrophotometer (IMPLEN, CA, USA) and an Agilent Bioanalyzer 2100 system (Agilent Technologies, CA, USA) and high-quality samples were used. Nine libraries for samples from three-time points were prepared using NEBNext ® UltraTM RNA library preparation kit for Illumina® (NEB, USA) following the manufacturer’s protocol. Briefly, the mRNA was purified using a poly-T oligo-attached magnetic beads. First strand cDNA was synthesized using random hexamer primers and M-MuLV reverse transcriptase and the second strand with DNA polymerase I and RNase H. Overhangs were converted into blunt ends by exonuclease/polymerase activity. To select cDNA fragments of preferentially 150–200 bp in length, the library fragments were purified with AMPure XP system (Beckman Coulter, Beverly, USA), which were then adapter ligated and incubated at 37 °C for 15 minutes followed by 5 minutes at 95 °C with 3 μl USER enzyme (NEB, USA). PCR with Phusion high-fidelity DNA polymerase, universal PCR primers, and index (X) primers was carried out and products were purified and quality assessed on Agilent Bioanalyzer 2100. Nine libraries with an average insert size of 175 bp were sequenced using the illumina HiSeq platform and paired-end reads were generated. The reference transcriptome sequences are available in NCBI’s SRA database with accession number SRP136574.

### *De Novo* transcriptome assembly and functional annotation

Raw data were filtered by removing the adaptor sequences, reads containing ploy-N and low-quality reads and contaminants of viral genes. *De novo* assembly of the clean reads was carried out with Trinity^58^ with minimum Kmer coverage set to 2 and all other parameters were set to default. Unigene’s functional annotation was done by BLASTx search against NCBI non-redundant protein database (Nr), NCBI nucleotide sequence database (Nt) and Swiss-Prot database with the e-value threshold of 1e-5 and against euKaryotic Orthologous Groups of proteins (KOG) with an e-value threshold of 1e-3. Prediction of protein structure domain with Protein family database (Pfam) was done with hmmscan (HMMER v3.0 package), with an e-value threshold of 0.01, Blast2GO v2.5^59^ with an e-value threshold of 1e-6 was used for annotation against Gene Ontology database (GO) and the threshold value of 1e-10 with KAAS, KEGG automatic annotation server was used to annotate against Kyoto Encyclopedia of Genes and Genomes (KEGG)^60^.

### Differential Expression analysis

Quantification of gene-level expression was estimated by RSEM^61^; DESeq R package was used for differential expression analysis between every two conditions. The *p*-values were adjusted using the Benjamini and Hochberg’s approach for controlling the false discovery rate. Genes with an adjusted *p*-value < 0.05 were assigned as differentially expressed.

### Gene set enrichment analysis and enrichment map

The biological context of genes and their enriched pathways were analyzed using the ranked gene set enrichment analysis (GSEA)^62^ and functional map was produced through enrichment map^63^ in cytoscape^64^. Our analysis is based on the heuristic Benjamini and Hochberg’s^65^ false discovery rate (FDR) procedure for enrichment analysis of gene expression data developed by Subramanian, *et al*.^62^. This procedure assumes that the challenge in estimating FDR because of likely involvement of the same gene in multiple gene sets can be overcome by performing sufficient permutations of case-control status (e.g., 1000 permutations) and re-analysis of the data under the null hypothesis^21,62^.

GO (Biological process, Molecular function, and Cellular component) and KEGG databases were used as a source for deriving gene sets. Terms annotating more than 1,500 or less than 5 genes were discarded because the large gene sets often represent too broad categories to derive useful biological meaning and too small gene sets are not likely to give statistically meaningful results. Groups of related treatments comparison were built using CK_3d, NPV_3d, and NPV_6d as low, higher and highest-responder phenotypes respectively. Thus, the comparison groups included the following combinations: NPV_3d versus CK_3d, NPV_6d versus NPV_3d and NPV_6d vs CK_3d. Differentially regulated genes (*P*_adj_ < 0.05 as determined by DESeq analysis) in those comparisons, were then perceived as the list of interesting genes. Rank scores were calculated as –log10 (*P*-value) multiplied by the sign of DESeq fold change such that upregulated genes had positive scores and downregulated had negative scores according to Debski, *et al*.^66^. The GSEA pre-ranked method based on 1000 gene sets permutations and default FDR of <0.25 were used for the subsequent analysis with automatic clustering and annotation through AutoAnnotate (v.1.2)^67^.

Gene sets enriched in the GSEA were organized into a graphical network produced using “Enrichment Map”^63^ plugins in Cytoscape (v.3.5.1)^64^. Plugin and source code are available at http://baderlab.org/Software/EnrichmentMap. Each node in the map represents an enriched gene set, which maps to a specific biological process/pathway and edges represent cross talks or gene overlap between the connected sets. Nodes are divided into three equal parts each representing the enrichment of comparison groups (figure legend in Fig. 3, 4 and Supplementary Fig. S7). Node color encodes the normalized enrichment scores (NES) calculated as Equation 1.

1 $${\rm{NES}}=\frac{{\rm{Actual}}\,{\rm{enrichment}}\,{\rm{score}}}{\mathrm{Mean}\,\,({\rm{Enrichment}}\,{\rm{scores}}\,{\rm{against}}\,{\rm{all}}\,{\rm{permutations}}\,\mathrm{of}\,\mathrm{the}\,{\rm{dataset}})}$$

Node size is proportional to the total number of genes belonging to the gene set. Edges were calculated with the overlapping coefficient of >0.5. The thickness of the edge is proportional to the number of shared genes between nodes. Each node is divided into three equal parts the first section in the clockwise direction from the top represents the enrichment obtained from NPV_3d vs CK_3d, the following section in the same direction represents the enrichment obtained for NPV_6d vs NPV_3d and the last section represents enrichment for NPV_6d vs CK_3d. The functional map is laid in organic layout and is clustered using AutoAnnotate plugin (v.1.2)^67^. 16 different clusters were produced which were identified by shaded ovals in the map. Clusters were named automatically by the clustering software and were manually edited for language clarification; the font size of the cluster label is proportional to the cluster size. Area of the cluster in the map does not represent any statistics and is for the sole purpose of easy visualization. Each cluster represented groups of enriched gene sets with overlapping gene sets and functional similarity.

The signaling pathway cluster was further extracted and presented in degree sorted circular layout for effective visualization and analysis of nodes and their interaction. Highly dense edges were sorted at overlap coefficient of >0.6 for easy visualization.

### RNA-Seq validation with quantitative RT-PCR

To validate the expression of unigenes obtained from RNA-Seq and to further analyze the reliability of RNA-Seq data, quantitative RT-PCR was conducted for 9 DEGs using 2xSYBR-green real time PCR mix (Takara) following manufacturer’s instruction. Total RNA was extracted from the samples with three biological replicates as that used for RNA-Seq. The Rotor-Gene Q Real-Time thermal cycler (Qiagen) was used to perform qPCR with 95 °C for 3 minutes, followed by 40 cycles of 95 °C for 15 seconds and 60 °C for 20 seconds. A melting curve was obtained from 60 to 90 °C with a 0.5 °C rise in temperature each 5 seconds to test the specificity of the amplified products. For normalization purpose, we selected reference gene Glyceraldehyde-3-phosphate dehydrogenase (*gapdh*) which showed no differential expression between samples according to the RNA-Seq data. The relative expression was calculated by 2^−ΔΔCT^^68^. The sequence of primers used in this study was designed according to sequence information obtained from RNA-Seq using Primer blast^69^ and is provided in Table S2.

### Sample preparation and analysis of circadian genes

Mock and virus-infected third instar gypsy moth larvae prepared as above were sampled every four hours from *Zeitgeber* time (ZT) 0 till 24 hours during 3 and 6-dpi (same days of sampling for RNA-Seq analysis). Samples were snap frozen in liquid nitrogen and stored at −80 °C until further analysis. Total RNA was extracted and quality assayed as above. First strand cDNA was synthesized from 1μg total RNA with oligoT per 20μl reaction using TransScript II First Strand cDNA Synthesis SuperMix (Takara, China). Prior to cDNA synthesis, RNA samples were treated with the gDNA eraser (Takara, China) to remove genomic DNA contamination. The qPCR was performed to examine the expression profiles of *per* and *tim* genes with *gapdh* as a reference gene in Rotor-Gene Q Real-Time thermal cycler (Qiagen). The PCR was performed in triplicates using following cycling parameters: 95 °C for 5 minutes, followed by 40 cycles of 15 seconds at 95 °C, 55 °C for 20 seconds and 72 °C for 20 seconds followed by melt curve analysis from 60 to 95 °C with 0.5 °C rise in temperature each 5 seconds to test the specificity of the amplified products. The Primers used were designed from the homologous sequences of the target genes obtained from RNA-Seq results using Primer blast^69^ listed in Table S2. Samples were analyzed in triplicates and relative expression of target genes was calculated by 2^-ΔΔCT^ method^68^. Expression of housekeeping gene from each sample was used to normalize the expression of target gene in each sample, which was further calibrated with the lowest normalized CT value among the samples from the same treatment and day. To compare gene expression between different experimental groups (Control, 3-dpi, 6-dpi) along the time course (ZT0 to ZT20) we performed two-way ANOVA followed by Dunnett’s multiple comparison tests. Analyses were conducted using GraphPad Prism (v. 6.07) (GraphPad, La Jolla, CA, USA).

### Directional light bio-assay

Virus treated larvae as in locomotion assay were subjected to different directional light treatments, larvae with columns assigned individually were placed inside an opaque plastic tube of 20 cm diameter and 45 cm height, ends capped with the same plastic lid and placed inside a chamber with the environment similar to those were reared upon. Three different light treatments were applied using 15 W led lights from a distance of 30 cm from the column: light from the top (14 L: 10 D), light from the bottom (14 L: 10 D) and no light condition (0 L: 24 D).

Height at death was recorded at 7-dpi and death from baculovirus infection was confirmed by screening for liquefaction of the larvae after death as above and those did not die of the virus were excluded from the analysis. Data were analyzed using Kruskal Wallis test followed by Dunn’s multiple comparison tests in SAS JMP statistical program version 13.2.0 (SAS Institute Inc., Cary, NC, USA). Each of these experiments was conducted with three independent replicates.

## Electronic supplementary material

Supplementary Information
