# Supplementary material for: Phototransduction and circadian entrainment are the key pathways in the signaling mechanism for the baculovirus induced tree-top disease in the lepidopteran larvae
Source: Sci Rep. 2018 Dec 3;8:17528. doi: 10.1038/s41598-018-35885-4 (PMC6277413; doi:10.1038/s41598-018-35885-4)
Supplement: Supplementary file 1 — Supplementary Information [file 41598_2018_35885_MOESM1_ESM.pdf]

Phototransduction and circadian entrainment are the key pathways in the signaling mechanism for the baculovirus induced tree-top disease in the lepidopteran larvae

Upendra Raj Bhattarai, Fengjiao Li, Mandira Katuwal Bhattarai, Abolfazl Masoudi, Dun Wang *

State Key Laboratory of Crop Stress Biology for Arid Areas, Northwest A&F University, Yangling, Shaanxi, 712100, P. R. China

*corresponding author wanghande@yahoo.com, dunwang@foxmail.com

Supplementary Information

Supplementary Table S1. Annotation summary of unigenes from illumine sequencing data to different databases.

Databases	Number of Unigenes	Percentage (%)
Annotated in NR	38561	22.79
Annotated in NT	21610	12.77
Annotated in KO	17632	10.42
Annotated in SwissProt	36057	21.31
Annotated in PFAM	41730	24.66
Annotated in GO	41898	24.76
Annotated in KOG	26371	15.58
Annotated in all Databases	5408	3.19
Annotated in at least one Database	61053	36.08
Total Unigenes	169188	100

Supplementary Table S2. List of primers used in the study.

Name of Primers	Sequence (5'-3')
JunF	TAAAGAGCACCGGGTCAGTC
JunR	CGTATGCGAGACGCAAGAAC
DdcF	GGACCGCTGGAGCTATTGAG
DdcR	CCGGCACATCTTATGCAAGC
GabaF	CTGCGAATTTTCTCGCCCTC
GabaR	AGCTGATTGCGTCATTGCTT
GlulF	GTTAGGTGAGTTCGCCAGCA
GlulR	ACTGCACGGGACAGACATAC
Myh6F	GGTAGCCTTGTCCTGCTCTG
Myh6R	GAAGGCTGACCTCGAGACAC

ActbF	CCATACCGACCATCACACC
ActbR	ACCACACACAAACGCCAAA
Hspa1F	TCCAGGTATTCGAGGGTGAG
Hspa1R	CCTTGTCGTTTGTGATGGTG
PepckF	GCGATCCACTTCGTACCATT
PepckR	TATCGTTTACGGCCTTTTGG
AntF	AGAGAGAGGCAACACCCTCA
AntR	TCGTCGTTGACTTCCTGATG
TimF	GGAGGGAGTCCGATGAGAGT
TimR	GTACCGTATCCGCAATCCGA
PerF	ACATTCTTGCGGGCGAAAAC
PerR	TTGGTTGGTAATCCGCTGCT
GapdhF	ACTGCCACCCAAAAGACTGT
GapdhR	CAGTGAGGTCGACAACGGAT

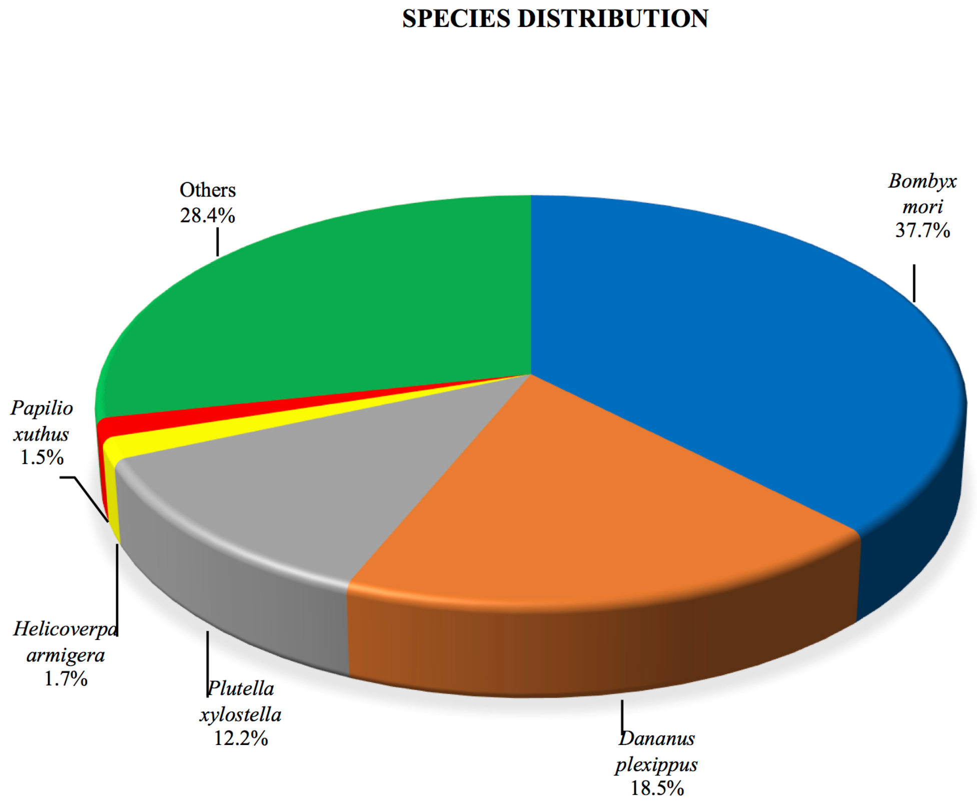

Supplementary Figure S1. Species distribution of the BLASTx results from the transcriptome annotations, numbers showed similarity percentage of genes to that species.

a

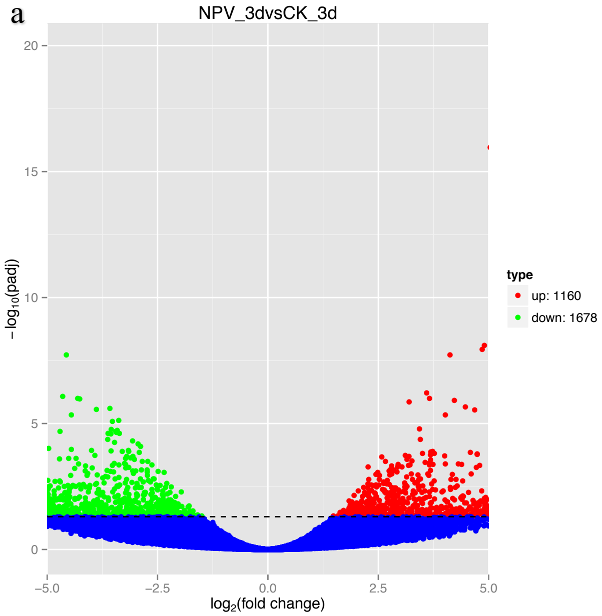

b

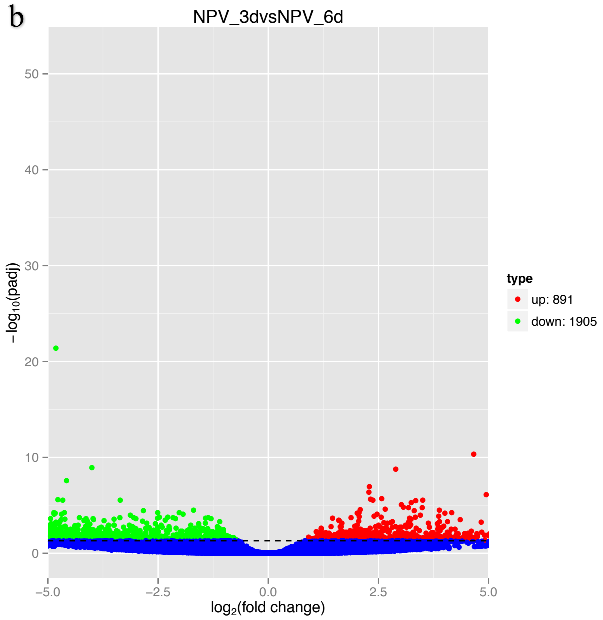

c

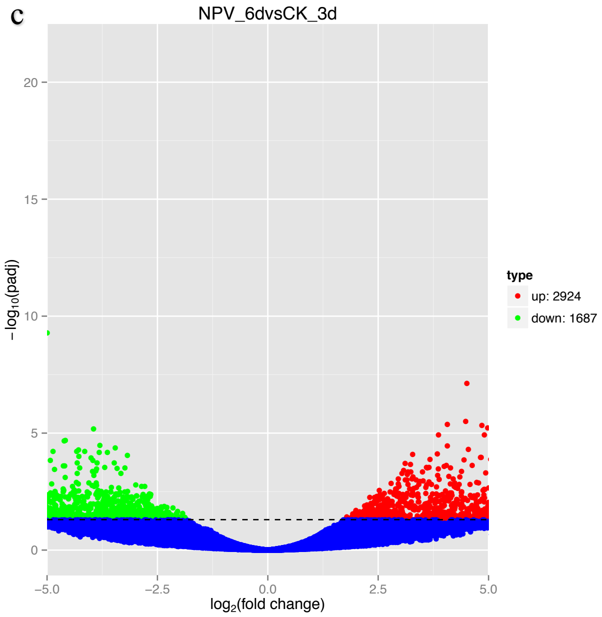

Supplementary Figure S2: Volcano plot from differential expression analysis showing the relative expression levels of transcripts in pairwise comparison of samples (a) NPV_3d vs CK_3d, (b) NPV_3d vs NPV_6d, (c) NPV_6d vs CK_3d. The x-axis represents the log2 of the expression fold change for each transcript. The y-axis represents the negative log10 of the adjusted *P*-value of the Fisher's Exact test. Red and green points represent statistically significantly (P_{adj}<0.05) up and down regulated genes respectively in pairwise comparisons.

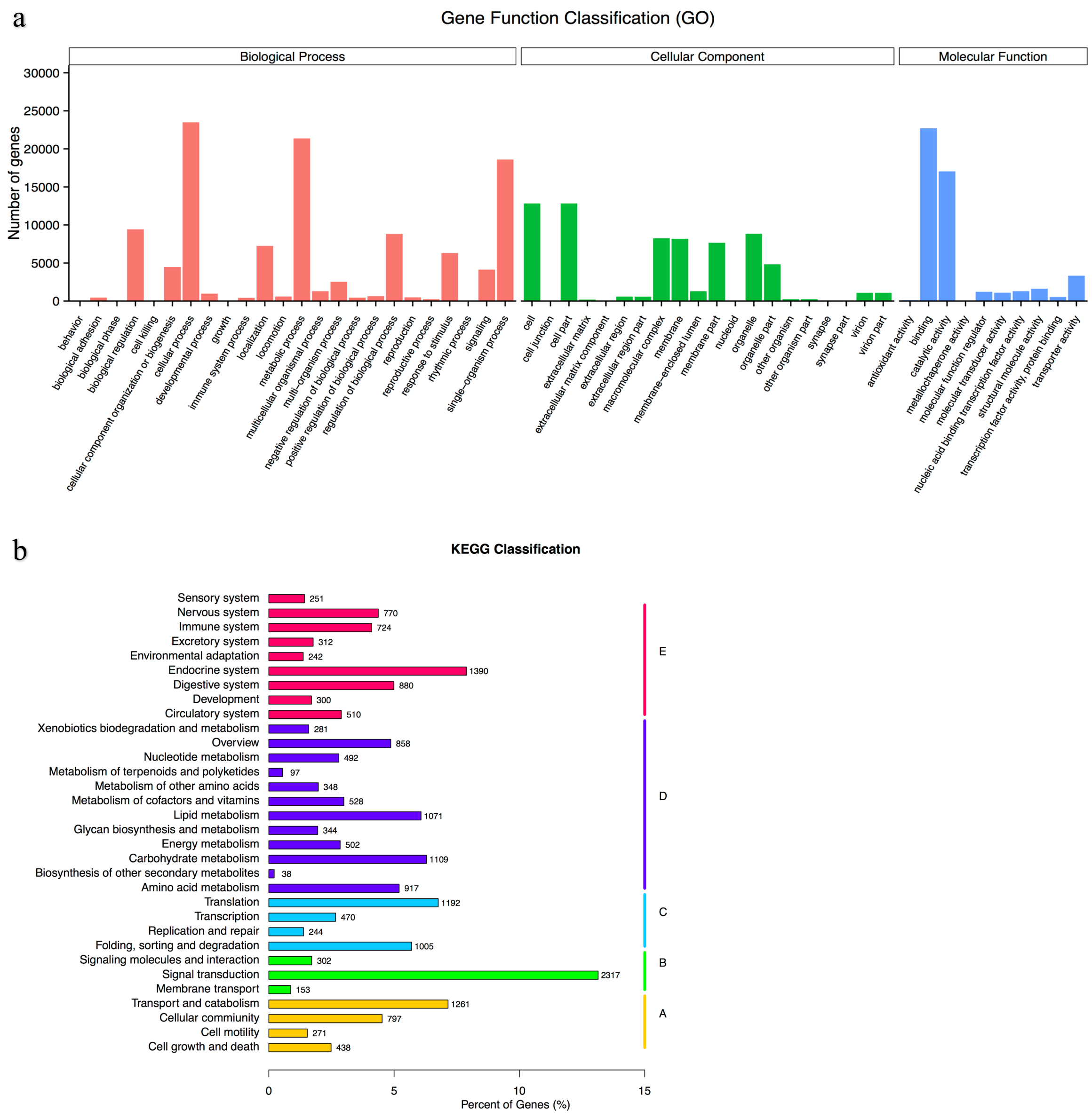

Supplementary Figure S3: Unigene's functional annotation and classification details. (a) Gene Ontology (GO) annotation and classification GO terms are summarized in three main categories of biological process, cellular component, and molecular function. Different GO terms are presented in X-axis; Y-axis represents the number of genes enriched. (b) KEGG annotation and pathway classification of unigenes, X-axis represents the percentage of genes involved. Left Y-axis is enlisted with different KEGG pathways, number of genes associated with each pathway are listed on top of each histogram. KEGG pathways are categorized in 5 different categories presented in right Y-axis: A stand for Cellular process category, B for Environmental Information processing, C for Genetic information processing, D for Metabolism and E for Organismal system category.

a

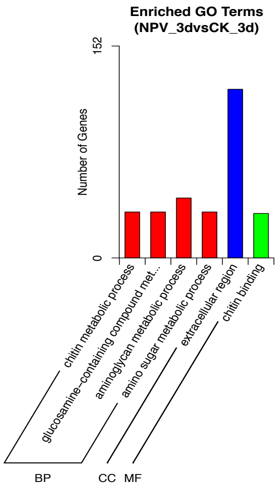

Supplementary Figure S4. Gene Ontology (GO) term distribution of the GO enrichment analysis of differentially expressed genes between samples (a) NPV_3d vs CK_3d; (b) NPV_3d vs NPV_6d; (c) NPV_6d vs CK_3d. GO terms are shown within the divisions of Biological Process (BP), Cellular Component (CC), and Molecular Function (MF) categories. Column height represents the number of enriched unigenes for the GO term.

b

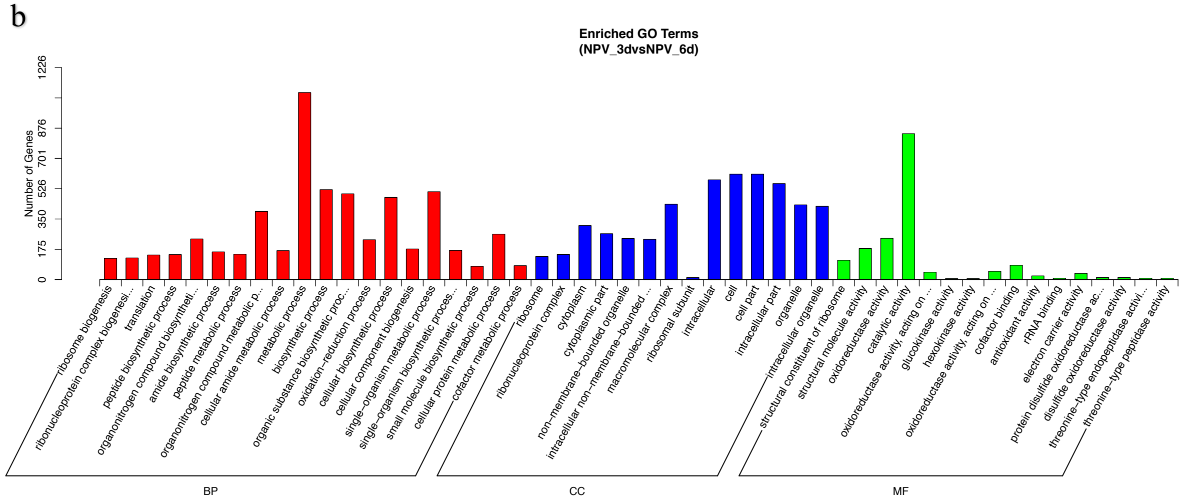

c

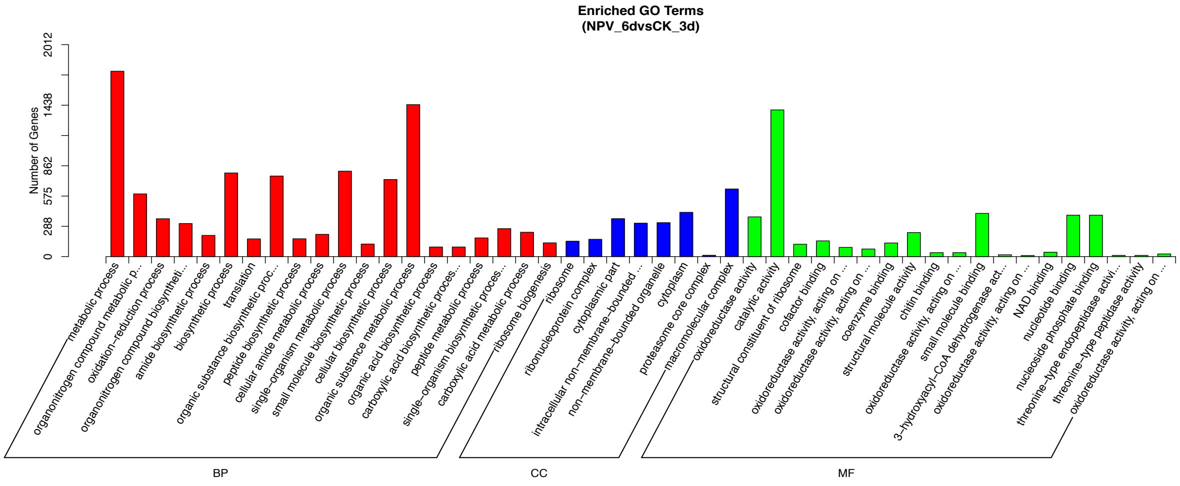

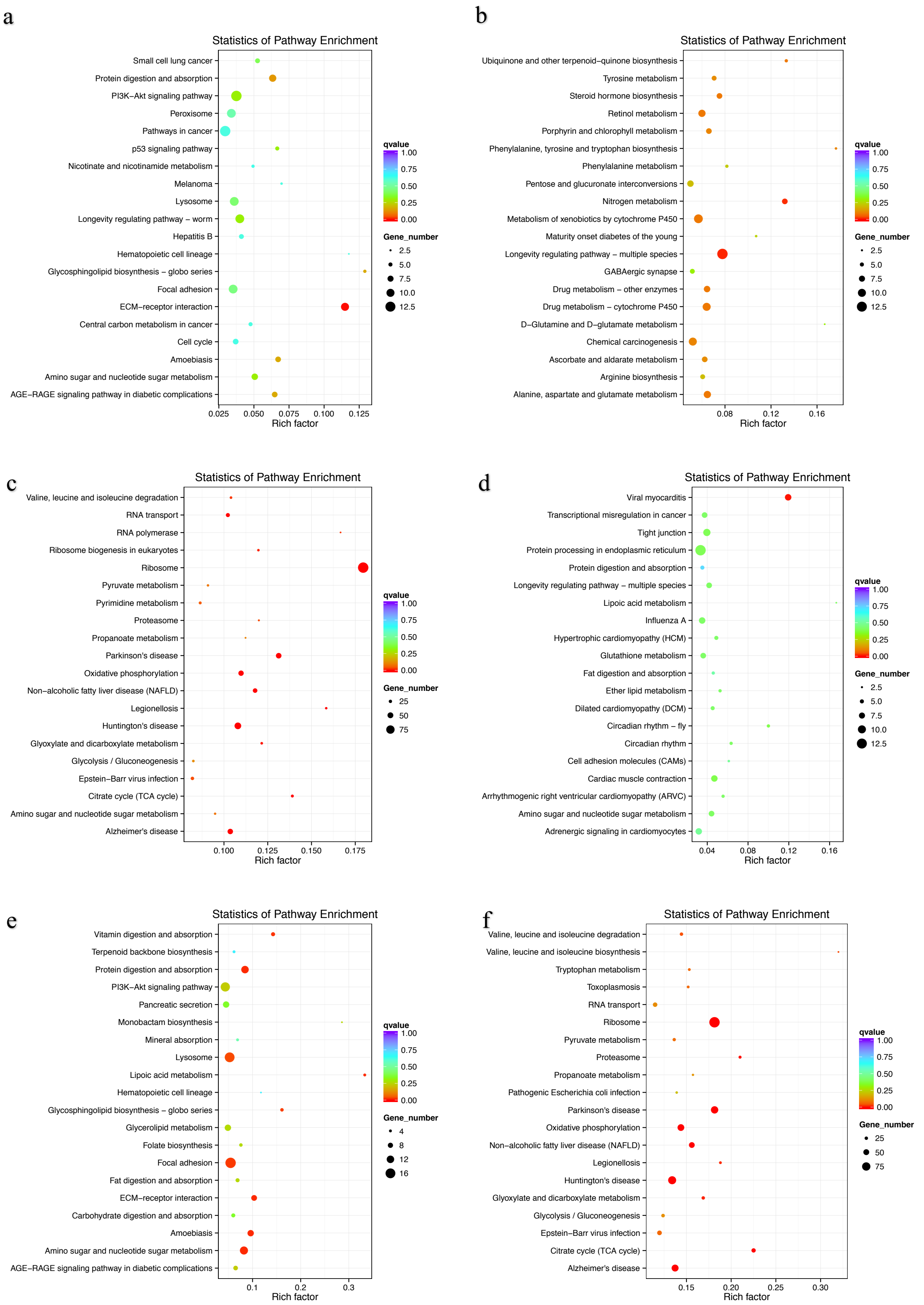

a
Statistics of Pathway Enrichment
Small cell lung cancer
Protein digestion and absorption
PI3K−Akt signaling pathway
Peroxisome
Pathways in cancer
p53 signaling pathway
Nicotinate and nicotinamide metabolism
Melanoma
Lysosome
Longevity regulating pathway − worm
Hepatitis B
Hematopoietic cell lineage
Glycosphingolipid biosynthesis − globo series
Focal adhesion
ECM−receptor interaction
Central carbon metabolism in cancer
Cell cycle
Amoebiasis
Amino sugar and nucleotide sugar metabolism
AGE−RAGE signaling pathway in diabetic complications
0.025 0.050 0.075 0.100 0.125
Rich factor
qvalue
1.00 0.75 0.50 0.25 0.00
Gene_number
2.5 5.0 7.5 10.0 12.5
b
Statistics of Pathway Enrichment
Ubiquinone and other terpenoid−quinone biosynthesis
Tyrosine metabolism
Steroid hormone biosynthesis
Retinol metabolism
Porphyrin and chlorophyll metabolism
Phenylalanine, tyrosine and tryptophan biosynthesis
Phenylalanine metabolism
Pentose and glucuronate interconversions
Nitrogen metabolism
Metabolism of xenobiotics by cytochrome P450
Maturity onset diabetes of the young
Longevity regulating pathway − multiple species
GABAergic synapse
Drug metabolism − other enzymes
Drug metabolism − cytochrome P450
D−Glutamine and D−glutamate metabolism
Chemical carcinogenesis
Ascorbate and aldarate metabolism
Arginine biosynthesis
Alanine, aspartate and glutamate metabolism
0.08 0.12 0.16
Rich factor
qvalue
1.00 0.75 0.50 0.25 0.00
Gene_number
2.5 5.0 7.5 10.0 12.5
c
Statistics of Pathway Enrichment
Valine, leucine and isoleucine degradation
RNA transport
RNA polymerase
Ribosome biogenesis in eukaryotes
Ribosome
Pyruvate metabolism
Pyrimidine metabolism
Proteasome
Propanoate metabolism
Parkinson's disease
Oxidative phosphorylation
Non−alcoholic fatty liver disease (NAFLD)
Legionellosis
Huntington's disease
Glyoxylate and dicarboxylate metabolism
Glycolysis / Gluconeogenesis
Epstein−Barr virus infection
Citrate cycle (TCA cycle)
Amino sugar and nucleotide sugar metabolism
Alzheimer's disease
0.100 0.125 0.150 0.175
Rich factor
qvalue
1.00 0.75 0.50 0.25 0.00
Gene_number
25 50 75
d
Statistics of Pathway Enrichment
Viral myocarditis
Transcriptional misregulation in cancer
Tight junction
Protein processing in endoplasmic reticulum
Protein digestion and absorption
Longevity regulating pathway − multiple species
Lipoic acid metabolism
Influenza A
Hypertrophic cardiomyopathy (HCM)
Glutathione metabolism
Fat digestion and absorption
Ether lipid metabolism
Dilated cardiomyopathy (DCM)
Circadian rhythm − fly
Circadian rhythm
Cell adhesion molecules (CAMs)
Cardiac muscle contraction
Arrhythmogenic right ventricular cardiomyopathy (ARVC)
Amino sugar and nucleotide sugar metabolism
Adrenergic signaling in cardiomyocytes
0.04 0.08 0.12 0.16
Rich factor
qvalue
1.00 0.75 0.50 0.25 0.00
Gene_number
2.5 5.0 7.5 10.0 12.5
e
Statistics of Pathway Enrichment
Vitamin digestion and absorption
Terpenoid backbone biosynthesis
Protein digestion and absorption
PI3K−Akt signaling pathway
Pancreatic secretion
Monobactam biosynthesis
Mineral absorption
Lysosome
Lipoic acid metabolism
Hematopoietic cell lineage
Glycosphingolipid biosynthesis − globo series
Glycerolipid metabolism
Folate biosynthesis
Focal adhesion
Fat digestion and absorption
ECM−receptor interaction
Carbohydrate digestion and absorption
Amoebiasis
Amino sugar and nucleotide sugar metabolism
AGE−RAGE signaling pathway in diabetic complications
0.1 0.2 0.3
Rich factor
qvalue
1.00 0.75 0.50 0.25 0.00
Gene_number
4 8 12 16
f
Statistics of Pathway Enrichment
Valine, leucine and isoleucine degradation
Valine, leucine and isoleucine biosynthesis
Tryptophan metabolism
Toxoplasmosis
RNA transport
Ribosome
Pyruvate metabolism
Proteasome
Propanoate metabolism
Pathogenic Escherichia coli infection
Parkinson's disease
Oxidative phosphorylation
Non−alcoholic fatty liver disease (NAFLD)
Legionellosis
Huntington's disease
Glyoxylate and dicarboxylate metabolism
Glycolysis / Gluconeogenesis
Epstein−Barr virus infection
Citrate cycle (TCA cycle)
Alzheimer's disease
0.15 0.20 0.25 0.30
Rich factor
qvalue
1.00 0.75 0.50 0.25 0.00
Gene_number
25 50 75

Supplementary Figure S5. Scatterplot of KEGG Pathway enrichment analysis. (a) NPV_3d vs CK_3d down regulated; (b) NPV_3d vs CK_3d up regulated; (c) NPV_3d vs NPV_6d down regulated; (d) NPV_3d vs NPV_6d up regulated; (e) NPV_6d vs CK_3d down regulated; (f) NPV_6d vs CK_3d up regulated. The degree of KEGG enrichment is measured by Rich factor, q-value and the number of genes enriched in this pathway. Rich factor refers to the ratio of the DEGs number in the pathway and the number of all genes annotated in the pathway. q-value is the *P*-value after normalization and its range is (0,1). The top20 significantly DEGs enriched pathways are displayed in the report. If the enriched pathways are less than 20, all enriched pathways are displayed.

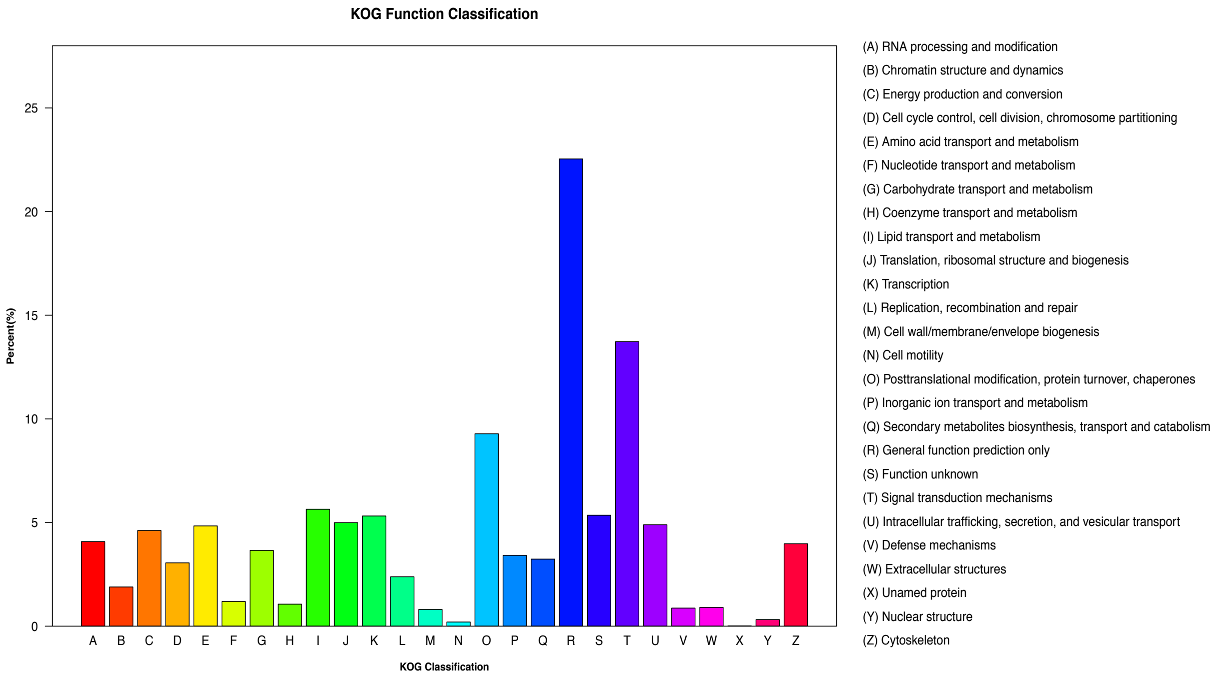

Supplementary Figure S6. KOG functional classification of *L. dispar* transcriptome. Y-axis represents percentage of unigenes enrichment and X-axis denotes different categories of KOG functional groups.

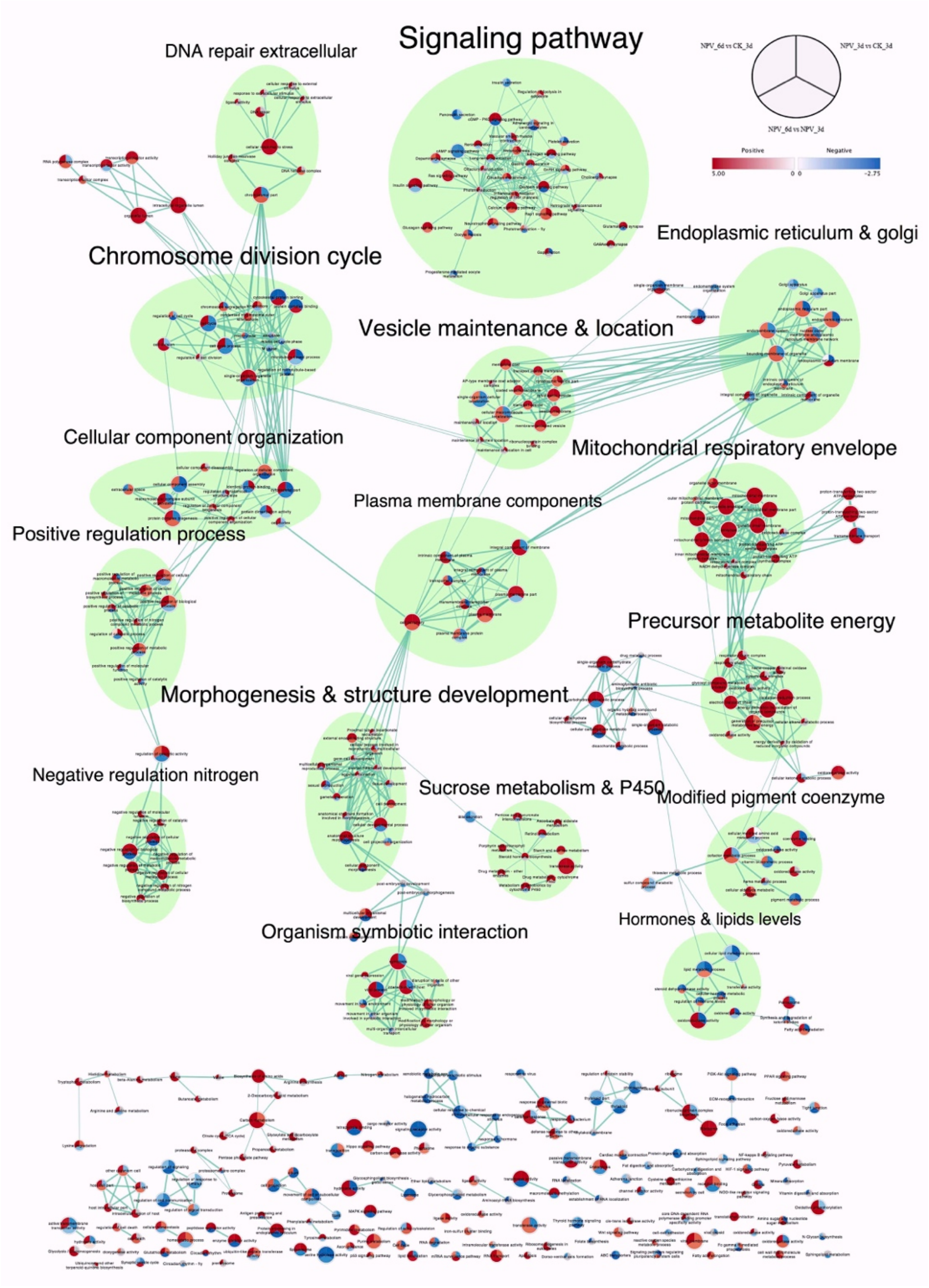

Supplementary figure S7. Complete enrichment Map visualization of the ranked GSEA results obtained for comparative analysis between each permutation of CK_3d, NPV_3d and NPV_6d, clustered and annotated by auto-annotate plugin, font size of the cluster label is proportional to the size of the cluster. Each node represents enriched GO/KEGG term, node size signifying the number of genes involved and is divided into three parts representing each one to one comparison between samples as illustrated in legend, Node color gradient represents the normalized enrichment scores (NES). Thickness of edges represents the richness of shared genes between connected nodes.

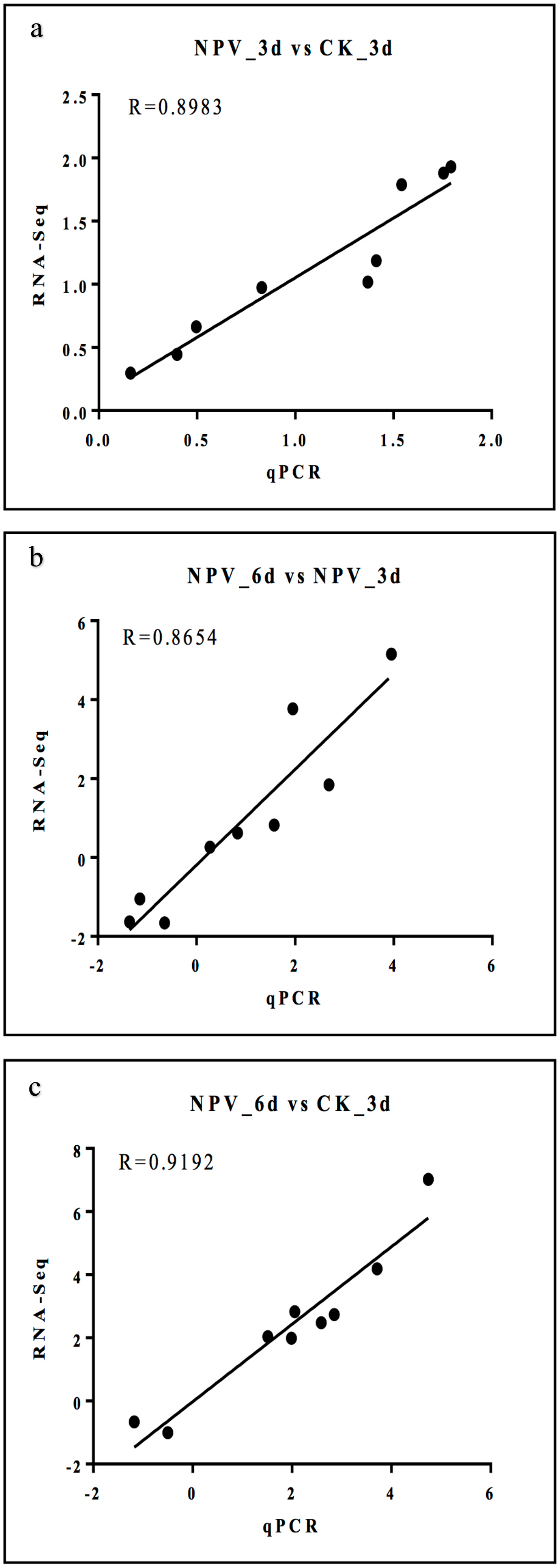

Supplementary Figure S8. Validation of RNA-Seq data analysis by RT-qPCR. The nine genes differentially expressed between CK_3d, NPV_3d and NPV_6d in pairs were again tested with RT-qPCR. (a) NPV_3d vs CK_3d; (b) NPV_6d vs NPV_3d; (c) NPV_6d vs CK_3d. Overall, expression patterns showed consistency between the two methods.

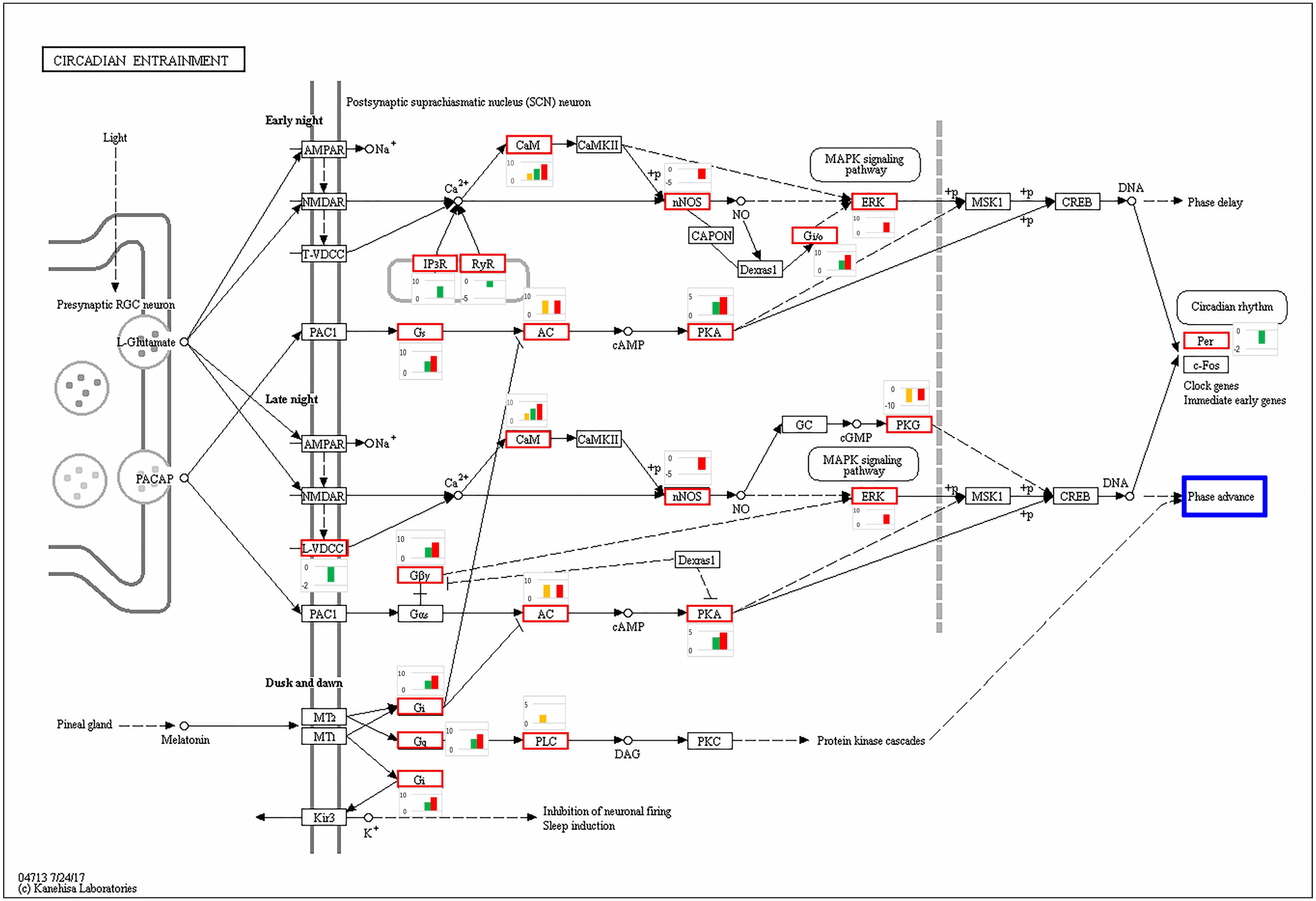
CIRCADIAN ENTRAINMENT
Light
Presynaptic RGC neuron
L-Glutamate
PACAP
Pineal gland
Melatonin
Postsynaptic suprachiasmatic nucleus (SCN) neuron
Early night
Late night
Dusk and dawn
AMPAR
NMDAR
T-VDCC
L-VDCC
PAC1
MT2
MT1
Kir3
Na+
Ca2+
K+
CaM
CaMKII
IP3R
RyR
Gs
Gαs
Gβγ
Gi
Gq
Gi/o
AC
PLC
PKA
PKC
PKG
GC
cAMP
cGMP
DAG
nNOS
NO
CAPON
Dexras1
ERK
MAPK signaling pathway
MSK1
CREB
+p
DNA
Phase delay
Phase advance
Circadian rhythm
Per
c-Fos
Clock genes
Immediate early genes
Protein kinase cascades
Inhibition of neuronal firing
Sleep induction
04713 7/24/17
(c) Kanehisa Laboratories

Supplementary figure S9. Circadian Entrainment pathway from KEGG database (https://www.kegg.jp/kegg/pathway/map/map04713.html), reference image; highlighting significantly differentially expressed genes. Nodes with red boxes are differentially expressed in pair wise comparison of samples, adjacent bar graph shows the expression pattern with column height representing the Log2FC, yellow column represents comparisons between NPV_3d vs CK_3d, green column for NPV_6d vs NPV_3d and red column for NPV_6d vs CK_3d. Phase advance of circadian cycle during treetop disease as seen our experiment is highlighted with blue boarder.
